# Dianhydrogalactitol induces replication-dependent DNA damage in tumor cells preferentially resolved by homologous recombination

**DOI:** 10.1038/s41419-018-1069-9

**Published:** 2018-10-03

**Authors:** Beibei Zhai, Anne Steinø, Jeffrey Bacha, Dennis Brown, Mads Daugaard

**Affiliations:** 1 Vancouver Prostate Centre, Vancouver, BC V6H 3Z6 Canada; 2 Department of Urologic Sciences, University of British Columbia, Vancouver, BC V5Z 1M9 Canada; 3 DelMar Pharmaceuticals, Inc., Vancouver, BC V5Z 1K5 Canada; 4 DelMar Pharmaceuticals, Inc., Menlo Park, CA 94025 USA

## Abstract

1,2:5,6-Dianhydrogalactitol (DAG) is a bifunctional DNA-targeting agent causing N^7^-guanine alkylation and inter-strand DNA crosslinks currently in clinical trial for treatment of glioblastoma. While preclinical studies and clinical trials have demonstrated antitumor activity of DAG in a variety of malignancies, understanding the molecular mechanisms underlying DAG-induced cytotoxicity is essential for proper clinical qualification. Using non-small cell lung cancer (NSCLC) as a model system, we show that DAG-induced cytotoxicity materializes when cells enter S phase with unrepaired N^7^-guanine DNA crosslinks. In S phase, DAG-mediated DNA crosslink lesions translated into replication-dependent DNA double-strand breaks (DSBs) that subsequently triggered irreversible cell cycle arrest and loss of viability. DAG-treated NSCLC cells attempt to repair the DSBs by homologous recombination (HR) and inhibition of the HR repair pathway sensitized NSCLC cells to DAG-induced DNA damage. Accordingly, our work describes a molecular mechanism behind N^7^-guanine crosslink-induced cytotoxicity in cancer cells and provides a rationale for using DAG analogs to treat HR-deficient tumors.

## Introduction

Historical data from preclinical studies and clinical trials support anti-neoplastic effects of 1,2:5,6-dianhydrogalactitol (DAG) analogs in a variety of cancer types, including leukemia, brain, cervical, ovarian, and lung cancers^1–6^. In China, DAG is approved for the treatment of lung cancer^7^. Worldwide, lung cancer is the leading cause of cancer-related deaths. The 5-year relative survival rate for lung cancer is 15% for men and 21% for women. There are two major types of lung cancer, non-small cell lung cancer (NSCLC) and small cell lung cancer (SCLC). NSCLC accounts for 80–85% of all lung cancer and approximately 57% of newly diagnosed NSCLC patients present with stage IV metastatic disease. The median overall survival for patients with stage IV NSCLC is 4 months, and the 5-year survival rate is only 4%^8–10^. Brain metastases occur frequently in NSCLC patients, contributing to the poor prognosis of this disease^11^. Currently, the mainstay treatments of primary and metastatic NSCLC include surgery, radiation therapy, chemotherapy, and targeted therapies with monoclonal antibodies or tyrosine kinase inhibitors (TKIs) in patients exhibiting epidermal growth factor receptor mutations^12–15^. However, the outcome of NSCLC patients remains poor mainly due to acquired platinum-based chemotherapy and TKI treatment resistance^16^.

DAG is a small water-soluble molecule that readily crosses the blood-brain barrier (BBB) and accumulates in primary and secondary brain tumors^4,17^. Perhaps for that reason, DAG displays strong activity in animal models of metastatic NSCLC, including TKI-resistant NSCLC^18^. Informed by preclinical studies, DAG may have a therapeutic advantage as compared to other DNA crosslinking agents^3,5^.

Due to its ability to cross the BBB, DAG is currently being tested in patients with temozolomide (TMZ) refractory glioblastoma multiforme (GBM)^19,20^. A recently completed phase I/II clinical trial in adult refractory GBM patients established a well-tolerated dosing regimen of DAG and confirmed myelosuppression as the dose-limiting toxicity with complete reversion upon treatment termination^21^. However, despite encouraging preclinical and clinical data in NSCLC and GBM, timely advancement of DAG analogs toward the clinical arena is hampered by inadequate understanding of the molecular mechanisms responsible for DAG-mediated cytotoxicity in cancer cells. We therefore used NSCLC as a model system to investigate the mechanisms of cytotoxicity imposed by the clinical-grade DAG analog VAL-083^22^.

## Results

### Loss of lung cancer cell viability after DAG treatment

**Fig. 1:**
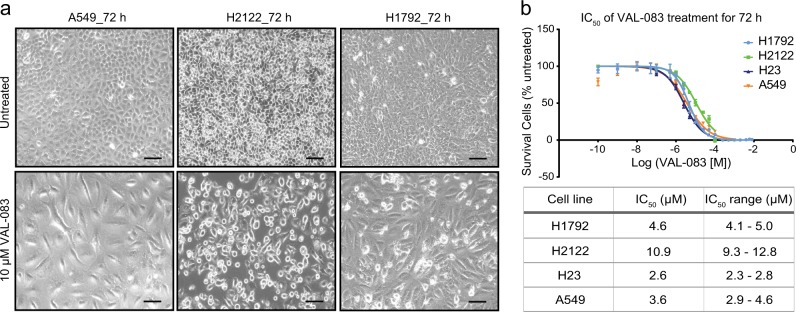
Cytotoxicity of DAG in NSCLC cell lines. **a** Bright-field images of A549, H2122, and H1792 cells cultured in 10 % FBS DMEM or RPMI 1640 medium for 72 h with or without 10 μM VAL-083 were shown. The scale bar represents 100 μm. **b** Four NSCLC cell lines A549, H23, H1792, and H2122 cells were seeded in 96-well culture plates and treated with different concentrations of VAL-083 (0, 100 nM, 500 nM, 1 μM, 2.5 μM, 5 μM, 10 μM, 25 μM, 50 μM, and 100 μM) for 72 h. Following the treatment, crystal violet assay was performed to detect the absorbance at 560 nm wavelength. The IC_50_ value of VAL-083 was determined by fitting a sigmoidal dose-response curve to the data using GraphPad Prism 6. The data on the curve are presented as mean ± standard error. Each cell line was tested in three to four individual experiments

To investigate the effects of DAG on lung cancer cells, we evaluated the cytotoxic activities of VAL-083 in a panel of NSCLC cell lines. Treatment of A549, H2122, and H1792 cells with 10 μM VAL-083 for 72 h resulted in dramatic morphological changes such as swelling and cell detachment (Fig. 1a). To further characterize the effect of DAG on tumor cells, we treated H1792, H2122, H23, and A549 NSCLC cell lines with different concentrations of VAL-083 for 72 h and subsequently determined viability of each cell line. The analysis showed a concentration-dependent loss of viability in all VAL-083-treated cell lines with half-maximal inhibitory concentration (IC_50_) values in the low µM concentration range (Fig. 1b). In summary, these data demonstrate cytotoxic effects of DAG on NSCLC cells.

### DAG induces persistent DNA damage in lung cancer cells

**Fig. 2:**
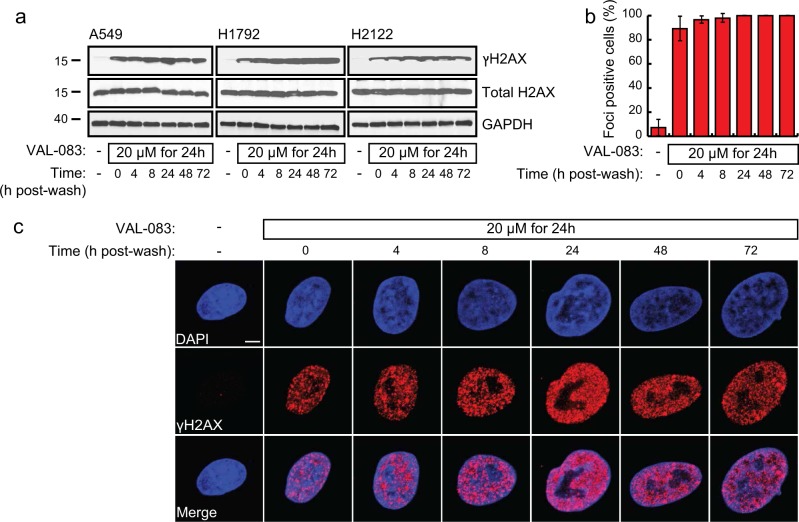
DAG induces DNA damage in NSCLC cells. **a** A549, H1792, and H2122 cells were treated with 20 μM VAL-083 for 24 h followed by washing and replacing with complete medium in culture for various periods of time (0, 4, 8, 24, 48, or 72 h). Then, cells were collected for protein extraction, and 50 μg was analyzed for phosphorylated and total H2AX expression by western blot using specific rabbit polyclonal antibodies as described under “Materials and methods”. Representative images are shown for the time-course effect of VAL-083 on ɣH2AX expression. Total H2AX and GAPDH served as loading controls. **b** Cultured A549 cells were treated with 20 μM VAL-083 for 24 h. After that, cells were washed and replaced with complete medium for an additional incubation time of 0, 4, 8, 24, 48, or 72 h. Then, cells were fixed, permeabilized, and immunostained with anti-ɣH2AX antibody. Quantification of ɣH2AX foci (cells with >10 foci were considered as “foci-positive”) from 40 to 50 cells per sample was shown. **c** Representative confocal images from each experimental conditions in **b** were shown with ɣH2AX in red. The scale bar represents 5 μm

Many chemotherapeutic drugs work by inducing different types of DNA damage in rapid-dividing cancer cells. DAG has been reported to have bifunctional DNA-targeting activity leading to the formation of N^7^-monoalkylguanine and inter-strand DNA crosslinks^22^. To investigate the effects of DAG on DNA integrity, we examined VAL-083-treated NSCLC cells for phosphorylated histone variant H2AX (ɣH2AX), an extensively used surrogate marker of DNA double-strand breaks (DSBs)^23,24^. Biochemical assessment of A549, H1792, and H2122 cells treated with VAL-083 for 24 h followed by removal of the drug showed strong ɣH2AX expression that persisted for 72 h after drug removal (Fig. 2a). This was supported by an immunofluorescence (IF) imaging analysis showing sustained ɣH2AX foci formation in 90–100 % of the cells (Fig. 2b) over a similar time frame (Fig. 2c). These data suggest that DAG induces DNA DSBs in NSCLC cells that cannot be repaired within a 72 h recovery period. In addition, VAL-083 induced strong ɣH2AX expression with at least 10 h incubation time (Supplementary Fig. S1a) and showed dose-dependent pattern (Supplementary Fig. S1b).

### Replication-dependent DNA damage of NSCLC cells upon DAG treatment

**Fig. 3:**
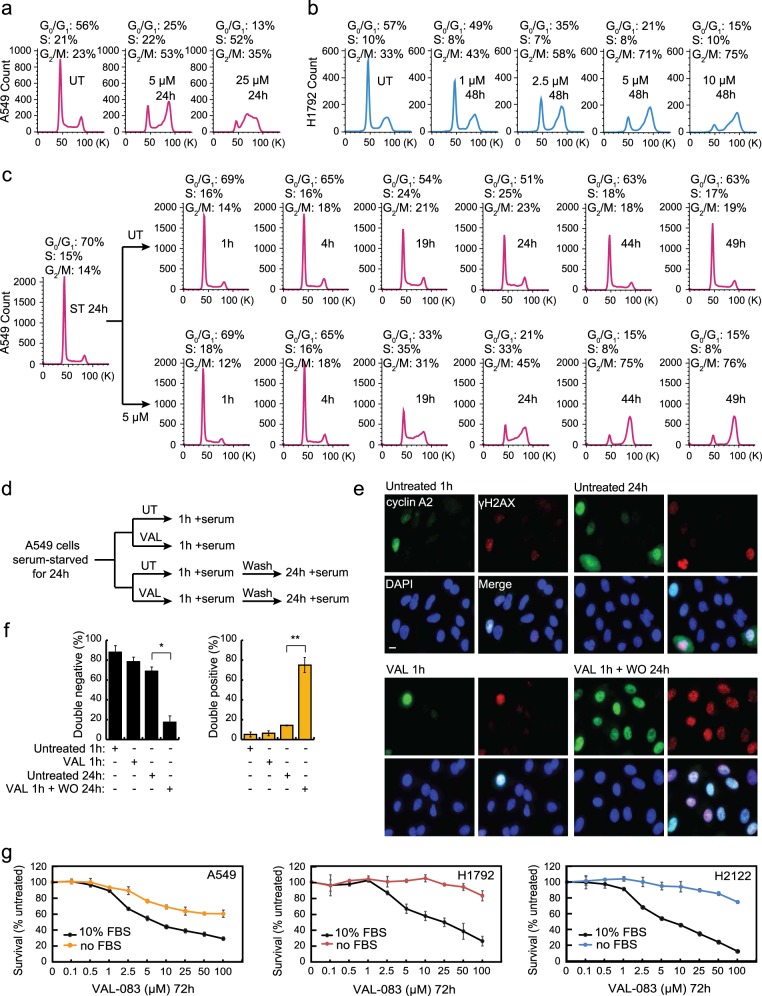
DAG-induced DNA damage occurs in S phase of the cell cycle. **a** A549 cells were treated with 5 or 25 μM VAL-083 for 24 h. After that, cells were collected for cell cycle analysis using PI staining by flow cytometry. **b** After treatment with different concentrations of VAL-083 (1, 2.5, 5, or 10 μM) in H1792 cells for 48 h, cell cycle analysis with PI staining was performed using flow cytometry. **c** A549 cells were synchronized by serum starvation (ST) for 24 h before treatment with or without 5 μM VAL-083 for the indicated time periods (1, 4, 19, 24, 44, or 49 h). Cells were then collected, fixed, and subsequently stained with PI for cell cycle distribution analysis by flow cytometry. For additional experimental details, see “Materials and methods”. The representative flow cytometric plots from two individual experiments are shown. **d** Flow chart outlining the experimental conditions used in **e**. **e** A549 cells (synchronized by 24 h serum starvation) were treated with 50 μM VAL-083 for 1 h and replaced with complete medium for another 24 h incubation. Then, cells were fixed, permeabilized, and immunostained with anti-cyclin A2 and anti-ɣH2AX antibodies. Representative IF images are shown with cyclin A2 in green and ɣH2AX in red. The scale bar represents 10 μm. **f** In all, 100–120 cells were examined from each treatment condition as in **e**. The percentages of two categories (cyclin A2−/ɣH2AX− and cyclin A2+/ɣH2AX+) of A549 cells are shown with corresponding statistical analysis (*p ≤ 0.05; **p ≤ 0.01; Student’s *t* test). **g** A549 cells were seeded with either serum-deprived or complete medium in 96-well culture plates. After 24 h incubation, cells were treated with 0, 100 nM, 500 nM, 1 μM, 2.5 μM, 5 μM, 10 μM, 25 μM, 50 μM, or 100 μM VAL-083 for 72 h. Following the treatment, the percentage of survival cells compared to the untreated condition was determined by crystal violet assay. The data are presented as mean ± standard error. H1792 and H2122 cells were also tested in the same experimental condition

We next investigated the effect of DAG on cell cycle progression using flow cytometric analysis of propidium iodide (PI)-stained NSCLC cells. VAL-083 treatment induced a strong dose-dependent S/G_2_-phase cell cycle arrest in A549 (Fig. 3a) and H1792 (Fig. 3b) NSCLC cells. This result suggests that DAG-mediated cytotoxicity likely depends on replication. To further determine the role of replication for DAG-induced cytotoxicity, we synchronized the majority of A549 cells in G_0_/G_1_ phase by serum starvation for 24 h (Fig. 3c). Cells were then released from the cell cycle arrest by addition of serum with or without 5 μM VAL-083 and followed by flow cytometry. While untreated cells rapidly assumed a normal cell cycle profile after serum addition, cells subjected to VAL-083 displayed a time-dependent S/G_2_ phase arrest visual at and after 19 h in complete medium (Fig. 3c). Notably, this concentration of VAL-083 treatment did not increase the sub-G_1_ population of cells (DNA content <2 N reflects cellular debris and apoptotic cells) indicating that the time-dependent decrease in G_0_/G_1_ was not due to increased cell death (Fig. 3c). In parallel western blot analysis of cleaved caspase 3 expression confirmed the lack of apoptosis in VAL-083-treated cells (Supplementary Fig. S2).

Cyclins and cyclin-dependent kinases (CDKs) are key regulators of eukaryotic cell cycle progression. Activation of cyclin B-Cdc2 kinase complexes trigger M-phase entry, whereas cyclin A-Cdk2 complexes control the progression through S phase^25–27^. Accordingly, cyclin expression is tightly regulated throughout the cell cycle where cyclin A is expressed exclusively in S phase plateauing in G_2_ phase^28^. Using cyclin A as a marker of S phase entry, we performed IF staining for cyclin A2 and ɣH2AX on A549 cells with or without VAL-083 pulse treatment (50 μM VAL-083 for 1 h) (Fig. 3d). The analysis showed that synchronized A549 cells displayed a dramatic accumulation of cyclin A2 (green) and ɣH2AX (red) expression after pulse treatment with VAL-083 followed by 24 h in complete medium (Fig. 3e). Importantly, the population of cells double-positive for cyclin A2+/ɣH2AX+ were significantly increased after VAL-083 pulse treatment for 1 h followed by washout for 24 h (VAL 1 h + WO 24 h) while double-negative cyclin A2−/ɣH2AX− cells decreased (Fig. 3f). This indicates that VAL-083-induced DNA N^7^-guanine crosslinks translate into DSBs in S/G_2_ phase of the cell cycle. To further validate the observed replication-dependent cytotoxicity of VAL-083 in NSCLC cell lines, we compared survival rates of cells cultured in complete medium with cells cultured in serum-deprived medium, challenged with increasing concentrations of VAL-083. Indeed, A549, H1792, and H2122 cells prohibited from entering S phase due to serum deprivation all showed resistance to VAL-083-induced cytotoxicity (Fig. 3g). Combined, these data suggest that DAG-induced cytotoxicity materializes as NSCLC cells progress into S phase of the cell cycle with unrepaired N^7^-guanine DNA crosslink lesions.

### DAG-induced DNA damage is preferentially repaired by homologous recombination

**Fig. 4:**
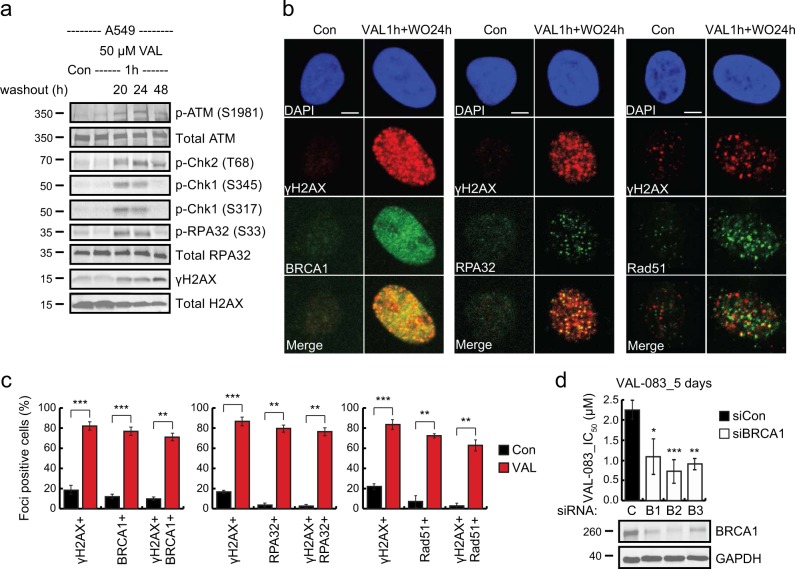
DAG-induced DNA damage is repaired by homologous recombination. **a** A549 cells were synchronized with 24 h serum starvation. After that, cells were incubated in complete medium with treatment of 50 μM VAL-083 for 1 h. Then, cells were washed and replaced with complete medium for an additional incubation time of 20, 24, or 48 h. Cell lysates were then extracted for western blot analysis of the sensors and effectors involved in the HR DNA damage response pathway using the following antibodies: phospho-ATM (Ser1981), phospho-Chk2 (Thr68), phospho-Chk1 (Ser345 and Ser317), phospho-RPA32 (Ser33), and ɣH2AX. Representative images are shown from three to four independent experiments. **b** A549 cells were synchronized with 24 h serum starvation. After that, cells were incubated in complete medium with treatment of 50 μM VAL-083 for 1 h. Then, cells were washed and replaced with complete medium for an additional incubation time of 24 h. Representative confocal images of indicated proteins (BRCA1, RPA32, Rad51, and ɣH2AX) were shown. The scale bar represents 5 μm. **c** Quantification of foci-positive A549 cells presented in **b** from 60–80 cells per condition were shown. Statistical analyses were obtained from three independent experiments (**p ≤ 0.01; ***p ≤ 0.001; Student’s *t* test). **d** A549 cells were transfected with either negative control (C) or three BRCA1-targeting siRNAs (B1, siBRCA1-2; B2, siBRCA1-15; or B3, siBRCA1-17) for 24 h. Cells were then seeded in 96-well culture plates and treated with different concentrations of VAL-083 (0, 100 nM, 500 nM, 1 μM, 1.5 μM, 2.5 μM, 5 μM, 10 μM, 25 μM, 50 μM, 100 μM, and 200 μM) for 5 days. Following the treatment, crystal violet assay was performed to detect the absorbance at 560 nm wavelength. The IC_50_ value of VAL-083 was determined by fitting a sigmoidal dose-response curve to the data using GraphPad Prism 6. The IC_50_ values of VAL-083 in control or BRCA1-knockdown cells are presented as mean ± standard error (*p ≤ 0.05; **p ≤ 0.01; ***p ≤ 0.001; Student’s *t* test). Cell lysates from the control or BRCA1-knockdown cells were analyzed by western blot with antibody against BRCA1, and GAPDH was used as a loading control. The representative images are shown from three independent experiments

As DAG induces DNA DSBs in S phase, cytotoxicity in cancer cells likely depends at least partially on their ability to repair DNA in S/G_2_ phase of the cell cycle. DNA DSBs can be repaired by either non-homologous end joining (NHEJ) or homologous recombination (HR). While NHEJ can occur throughout the cell cycle, HR is restricted to the S and G_2_ phases where sister chromatids are available as templates for sequence homology-guided repair^29^. Since DAG-induced DNA DSBs materialized specifically in S/G_2_, we hypothesized that NSCLC cells would repair these lesions by HR. To test this hypothesis, we monitored DNA DSB sensors and effectors involved in the HR pathway in NSCLC cells after VAL-083 treatment. VAL-083 pulse treatment followed by 20–48 h incubation in complete medium without VAL-083 triggered the activation of ataxia telangiectasia mutated (ATM) kinase in A549, H1792, and H2122 cells (Fig. 4a and Supplementary Fig. S3). This was associated with threonine 68 phosphorylation of S phase checkpoint kinase Chk2 and the presence of ɣH2AX. Upon activation of HR repair, the DNA surrounding the DSBs is processed in an enzymatic step that depends on CtIP and hepatoma-derived growth factor family co-factors to create a single-stranded DNA (ssDNA) template for homology pairing^30–33^. The resected ssDNA is subsequently occupied by ssDNA-binding replication protein A (RPA32) that is phosphorylated on serine 33 by the ataxia telangiectasia and Rad3-related protein (ATR) kinase in response to ssDNA exposure^34^. This event triggers the final step in the HR pathway where homology repair is completed in a Rad51-dependent manner^35^. Indeed, VAL-083 treatment induced phosphorylation of RPA32 as well as the Chk1 kinase, a well-known phospho-target of ATR (Fig. 4a). In aggregate, these data suggest that NSCLC cells repair DAG-induced DSBs by HR. To further substantiate this finding, we analyzed the recruitment of key HR repair proteins to ɣH2AX foci in NSCLC cells treated with VAL-083 by confocal microscopy. The analysis showed that HR repair proteins BRCA1, RPA32, and Rad51^35^ co-localized with ɣH2AX foci induced by VAL-083 (Fig. 4b) and these co-localizations were statistically significant (Fig. 4c). Combined, these data demonstrate that NSCLC cells repair DAG-induced DSBs by HR repair.

Our data entertain a prediction that cancer cells deficient in HR repair would have increased sensitivity to DAG. To confirm this prediction, we knocked down the essential HR repair protein BRCA1^36,37^ in NSCLC cells using small interfering RNAs (siRNAs) before treatment with VAL-083. Indeed, knockdown of BRCA1 using three non-overlapping siRNAs significantly sensitized A549 cells to VAL-083 (Fig. 4d). These data confirm the prediction that cancer cells deficient in HR repair are unable to resolve DAG-induced DSBs.

## Discussion

DNA crosslinking agents are widely used as chemotherapy in a variety of cancers^38^. While they all target DNA, there are small differences that distinguishes these agents from each other. For example, most agents have more than one function and it is often difficult to uncouple these functions in terms of mechanism of action. DAG is a bifunctional DNA-targeting agent causing N^7^-monoalkylguanine and inter-strand DNA crosslinks^22^, but it has also been reported to inhibit angiogenesis^7^. DAG has been shown to interact with DNA yielding 7-(1-deoxygalactit-1-yl)guanine, 7-(1-deoxyanhydrogalactit-1-yl)guanine, and 1,6-di(guanin-7-yl)-1,6-dideoxygalactitol, of which the last product indicates inter- or intra-strand crosslink formation^39^. Up to this date, more than 40 NCI-sponsored phase I and II clinical trials involving DAG has been conducted in the United States. Preclinical and clinical trial data suggest antitumor activity of DAG in several malignancies including lung cancer, brain tumors, leukemia, cervical cancer, and ovarian cancer^1–6^. Also, an open-label post-market clinical trial in China investigates the activity of VAL-083 in relapsed or refractory NSCLC patients^40^.

**Fig. 5:**
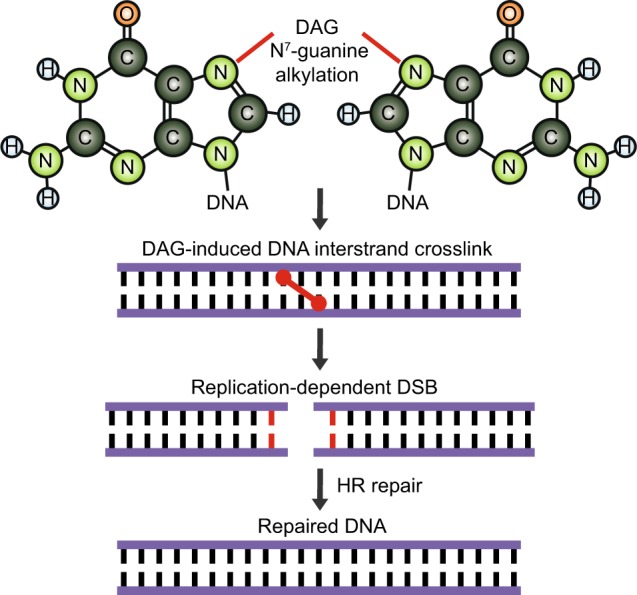
Model of the mechanism of action of DAG in lung cancer cells. DAG treatment induces inter-strand DNA crosslinks through N^7^-guanine alkylation, leading to replication-dependent DNA double-strand breaks (DSB). Lung cancer cells respond to the DAG-induced DNA DSB through activation of HR DNA repair

TMZ is a DNA alkylating agent targeting N^7^ and O^6^ positions of guanine and is currently used as first-line treatment of GBM^41^. Interestingly, while TMZ and DAG have at least partially overlapping properties in terms of DNA interactions, mechanisms of cytotoxicity appear to be different. For example, GBM cells resistant to TMZ still show sensitivity to DAG^42^, indicating non-overlapping functions between the two agents. Precise knowledge of the molecular mechanisms underlying tumor cell cytotoxicity is essential for optimal positioning of chemotherapeutic drugs in a clinical context. For that reason, we decided to dissect and describe the cytotoxic mechanisms of DAG using NSCLC as our model system. We found that VAL-083, a good manufacturing practice-produced clinical-grade DAG, is well-tolerated by cells as long as they are in G_1_ phase of the cell cycle. Cells have several systems in place that can resolve DNA crosslinks and methylations such as nucleotide excision repair (intra-strand) and the Fanconi anemia system (inter-strand)^38,43^. Repair of DNA crosslinks in G_1_ phase is supported by the G_1_/S phase checkpoint machinery that keeps cells in G_1_ until repair has been completed^44^. However, the G_1_/S phase checkpoint is often compromised in cancer cells and in this scenario, cells may enter S phase with unrepaired DNA lesions^45^. When the replication forks collide with the DNA lesions, replication is blocked exposing stretches of ssDNA that will subsequently be bound by RPA. The violent collision between the DNA lesion and the replication machinery will often result in DNA DSBs, which are catastrophic for the cells^46^. Indeed, we found the cytotoxic effect of DAG on NSCLC cells to develop in S phase indicating that these cells progress from G_1_ to S phase with unrepaired DNA lesions. In S phase, DAG-treated cells display a profound ɣH2AX response indicative of DNA DSBs. We furthermore found that these DAG-induced DSBs activated the HR pathway and inhibition of HR dramatically sensitized NSCLC cells to DAG. As such, our data suggest that DAG-induced DNA lesions translate into replication-dependent DNA DSBs in S phase that are subsequently repaired by HR (Fig. 5). Importantly, our work provides a clinical rationale for positioning DAG-based chemotherapy in cancers deficient in HR repair.

## Materials and methods

### Reagents and cell culture

VAL-083 was obtained from DelMar Pharmaceuticals, Inc. (Vancouver, Canada and Menlo Park, CA, USA). PI solution (1 mg/ml) and glutaraldehyde solution (grade I, 50% in H_2_O) were purchased from Sigma-Aldrich (Oakville, Canada). Sorenson’s solution was prepared with 9 mg trisodium citrate, 195 ml 0.1 N HCl, 500 ml 90% ethanol, and 305 ml distilled water. All cell lines were maintained at 37 °C in 5% CO_2_ atmosphere. A549 cells were cultured in Dulbecco’s modified Eagle’s medium supplemented with 10% fetal bovine serum. H2122, H1792, and H23 cells were cultured in RPMI 1640 with 10% fetal bovine serum.

### Crystal violet cell proliferation assay

Following 72 h of different concentrations of VAL-083 treatment, cells were fixed in 1% glutaraldehyde solution for 5 min. After rinsing with distilled water, cells were incubated with 0.1% crystal violet solution dye for 10 min. Cells were then gently washed with distilled water and air-dried. The crystals on the plate were dissolved in Sorenson’s solution before reading absorbance at 560 nm wavelength with a microplate reader. Survival cells were expressed as the percentage compared to untreated cells.

### Cell cycle analysis using PI staining

Cell cycle distribution was evaluated based on DNA content using PI staining. Serum starvation (24 h)-synchronized cells were treated with 5 μM VAL-083 for 1, 4, 19, 24, 44, and 49 h. Cells were then trypsinized, washed in phosphate-buffered saline (PBS), and centrifuged at 1000 rpm for 5 min. Cell pellets were fixed in 70% ethanol at least overnight at 4 °C. After washing with PBS, cells were incubated with 500 μl PI solution in PBS containing 50 μg/ml PI, 100 μg/ml RNase A, and 0.05% Triton X-100 for 40 min at 37 °C in the dark. Thereafter, cells were washed and resuspended in PBS. DNA content were analyzed by flow cytometry (FACS Canto II), and histograms and quantitative analyses of the proportions of cells in G_0_/G_1_, S and G_2_/M phases were made using FlowJo software. Untreated cells were included as control.

### Western blotting

Cells were lysed in EBC buffer (50 mM Tris-HCl, pH 8.0, 120 mM NaCl, 1% NP-40, and 1 mM EDTA) supplemented with phosphatase inhibitor and protease inhibitor (Roche, Mississauga, Canada). Cellular proteins were separated by SDS-polyacrylamide gel electrophoresis and transferred onto polyvinylidene fluoride membrane. After incubation with blocking buffer for 1 h, the membrane was incubated with designated primary antibodies overnight at 4 °C. Then, membrane was washed three times for 10 min with TBST and incubated with horseradish peroxidase-conjugated anti-mouse or anti-rabbit antibodies (Santa Cruz Biotechnology, Dallas, TX, USA) for 1–2 h. Membrane was washed with TBST three times and developed with Pierce ECL substrate system (ThermoFisher Scientific, Burlington, Canada) according to the manufacturer’s instruction. The following primary antibodies were used for immunoblotting: ɣH2AX (Cell Signaling Technology, Danvers, MA, USA, 2577); H2AX (Abcam, Toronto, Canada, ab11175); phospho-ATM (Ser1981) (Rockland Antibodies and Assays, Limerick, PA, USA, 200-301-400); ATM (Cell Signaling Technology, 2873); GAPDH (Cell Signaling Technology, 5174); phospho-RPA32 (Ser33) (Bethyl Laboratories, Montgomery, TX, USA, A300-246A); phospho-Chk1 (Ser345) (Cell Signaling Technology, 2348); phospho-Chk1 (Ser317) (Cell Signaling Technology, 12302); phospho-Chk2 (Thr68) (Cell Signaling Technology, 2661); RPA32 (Abcam, ab2175); BRCA1 (Novus Biologicals, Oakville, Canada, NB 100-404); and cleaved caspase 3 (Cell Signaling Technology, 9661). Representative blotting images were shown from three to four independent experiments.

### IF and microscope

Cells were grown on glass coverslips for at least 16 h before serum starvation for 24 h. Synchronized cells were treated with 50 μM VAL-083 for 1 h followed by washout and incubation with complete medium for another 24 h. Subsequently, cells were washed once with PBS and fixed for 30 min with 4% paraformaldehyde in PBS at room temperature. For DNA damage foci detection, cells were pre-extracted with cytoskeletal buffer (25 mM HEPES, pH 7.4, 50 mM NaCl, 1 mM EDTA, 3 mM MgCl_2_, 300 mM sucrose, and 0.5% Triton X-100) for 5 min at 4 °C before fixation with 4% paraformaldehyde solution. Fixed cells were washed three times with PBS and permeabilized for 20 min with 0.5% Triton X-100 in PBS. After washing with PBS for three times and blocking with 3% bovine serum albumin in PBS for 1 h at room temperature, cells were incubated overnight at 4 °C with corresponding primary antibodies diluted in blocking solution. The next day, cells were washed three times with PBS and incubated with appropriate fluorophore-labeled secondary antibodies for 1 h at room temperature. After washing with PBS for three times, the coverslips were mounted with Vectashield mounting medium (with 4′,6-diamidino-2-phenylindole). Images were acquired using Zeiss AxioObserver microscope and confocal LSM-780 microscope. The LSM-ZEN software was used for analyzing the images. The following primary and secondary antibodies were used in IF staining: ɣH2AX (Cell Signaling Technology, 2577); cyclin A2 (Abcam, ab16726); ɣH2AX (EMD Millipore, Etobicoke, Canada, 05-636); BRCA1 (Abcam, ab16780); Rad51 (Santa Cruz Biotechnology, H8349); RPA32 (Abcam, ab2175); donkey anti-rabbit Alexa-Fluor 594 (ThermoFisher Scientific, A21207); donkey anti-rabbit Alexa-Fluor 488 (ThermoFisher Scientific, A21206); donkey anti-mouse Alexa-Fluor 594 (ThermoFisher Scientific, A21203); and donkey anti-mouse Alexa-Fluor 488 (ThermoFisher Scientific, A21202). Representative images were shown from three independent experiments.

### siRNA transfection

A549 cells were transfected with either a control siRNA or siRNAs targeting BRCA1 using RNAiMAX transfection reagent (ThermoFisher Scientific) according to the manufacturer’s instruction. After 24 h of transfection, A549 cells were seeded in 96-well culture plates and treated with different concentrations of VAL-083 for 5 days followed by crystal violet assay. In parallel cell lysates were collected for western blot verification of BRCA1 knockdown. The siRNAs used in this study were as follows: C, siCon (negative control medium GC duplex, Invitrogen, 462001); B1, siBRCA1-2 (Qiagen, Toronto, Canada, SI00096313); B2, siBRCA1-15 (Qiagen, SI02664368); and B3, siBRCA1-17 (Qiagen, SI03103975).

### Statistical analysis

Where indicated, *p*-values were calculated using Student’s *t* test. Data were presented as mean ± SD of three independent experiments.

## Electronic supplementary material

Supplementary Figure Legends

Supplementary Figure S1

Supplementary Figure S2

Supplementary Figure S3
