# Supplementary material for: Dianhydrogalactitol induces replication-dependent DNA damage in tumor cells preferentially resolved by homologous recombination
Source: Cell Death Dis. 2018 Oct 3;9(10):1016. doi: 10.1038/s41419-018-1069-9 (PMC6170372; doi:10.1038/s41419-018-1069-9)
Supplement: Supplementary file 1 — Supplementary Figure Legends [file 41419_2018_1069_MOESM1_ESM.doc]

**Dianhydrogalactitol induces replication-dependent DNA damage in tumor cells preferentially resolved by homologous recombination**

Beibei Zhai^1, 2^, Anne Steinø^3^, Jeffrey Bacha^3^, Dennis Brown^3^, and Mads Daugaard^1, 2^, *

^1^ Vancouver Prostate Centre, Vancouver, BC, V6H 3Z6, Canada

^2^ Department of Urologic Sciences, University of British Columbia, Vancouver, BC, V5Z 1M9, Canada

^3^ DelMar Pharmaceuticals, Inc., Vancouver, BC, V5Z 1K5, Canada and Menlo Park, CA, 94025, USA

*To whom correspondence should be addressed: Mads Daugaard, Vancouver Prostate Centre, Vancouver, BC V6H 3Z6, Canada; Department of Urologic Sciences, University of British Columbia, Vancouver, BC V5Z 1M9, Canada. Tel: +1 604 875 4111 ext 21792; Fax: +1 604 875 5654; E-mail: [mads.daugaard@ubc.ca](mailto:mads.daugaard@ubc.ca)

**Running Title:** Mechanism of VAL-083 in lung cancer

**Supplementary figure legends:**

**Fig. S1: VAL-083 treatment induces ɣH2AX expression in time- and dose-dependent patterns**

**a** A549 and H1792 cells were treated with 20 μM VAL-083 in complete medium for the indicated periods of time (1 h, 2 h, 4 h, 10 h, 20 h, or 24 h). Cells were then collected for Western blot analysis of ɣH2AX, and total H2AX and GAPDH were included as loading controls.

**b** A549 and H1792 cells were treated with different concentrations of VAL-083 (0, 5 μM, 10 μM, 20 μM, 30 μM, 40 μM, or 50 μM) in complete medium for 24 h. Cells were then collected for Western blot analysis of ɣH2AX, and total H2AX and GAPDH were included as loading controls.

**Fig. S2: VAL-083 treatment does not trigger apoptotic cell death in A549 cells**

A549 cells were synchronized by serum starvation for 24 h before treatment with or without 5 μM VAL-083 in complete medium for the indicated periods of time (1 h, 4 h, 19 h, 24 h, 44 h or 49 h). Cells treated with 1 μM staurosporine (ST) for 5 h were used as a positive control for apoptosis. Cells were then collected for Western blot analysis of cleaved caspase 3, and GAPDH was used as a loading control.

**Fig. S3: VAL-083 treatment induces HR activation in H1792 and H2122 cells**

H1792 and H2122 cells were synchronized by 24 h serum starvation. After that, cells were incubated in complete medium with treatment of 50 μM VAL-083 for 1 h. Then, cells were washed and replaced with complete medium for an additional incubation time of 20 h, 24 h or 48 h. Cell lysates were then extracted for Western blot analysis of mediators involved in the HR DNA damage response pathway using the following antibodies: phospho-ATM (Ser1981), phospho-Chk2 (Thr68), phospho-Chk1 (Ser345 and Ser317), phospho-RPA32 (Ser33), and ɣH2AX. Representative images are shown from 3 - 4 independent experiments.
