# Supplementary figures and images for: Dianhydrogalactitol induces replication-dependent DNA damage in tumor cells preferentially resolved by homologous recombination
Source: Cell Death Dis. 2018 Oct 3;9(10):1016. doi: 10.1038/s41419-018-1069-9 (PMC6170372; doi:10.1038/s41419-018-1069-9)

Figure S1

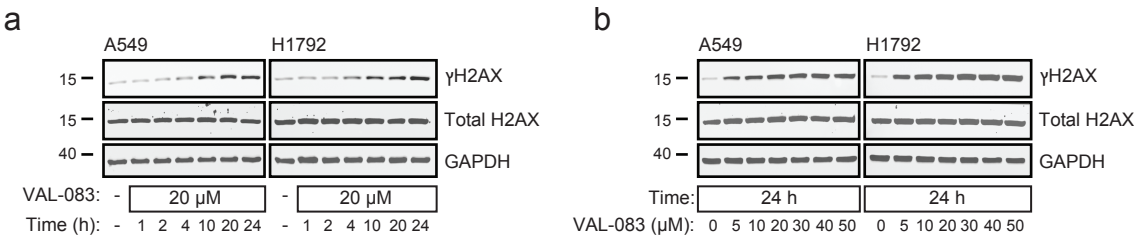

Supplement: Supplementary file 2 — Supplementary Figure S1 [file 41419_2018_1069_MOESM2_ESM.pdf]

Figure S2

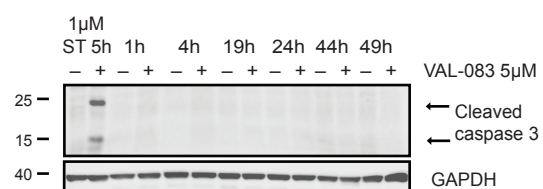

Supplement: Supplementary file 3 — Supplementary Figure S2 [file 41419_2018_1069_MOESM3_ESM.pdf]

Figure S3

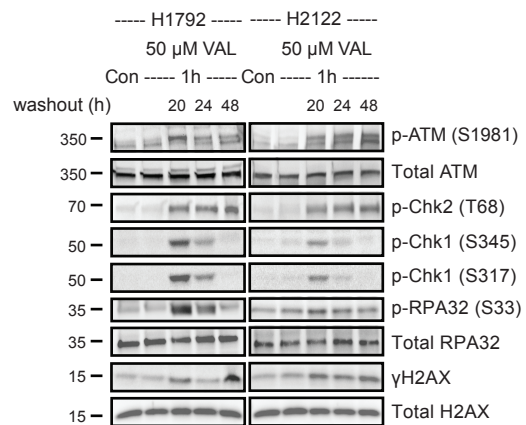

Supplement: Supplementary file 4 — Supplementary Figure S3 [file 41419_2018_1069_MOESM4_ESM.pdf]
